# Liquid Biopsy as a Tool for Differentiation of Leiomyomas and Sarcomas of Corpus Uteri

**DOI:** 10.3390/ijms20153825

**Published:** 2019-08-05

**Authors:** Dana Dvorská, Henrieta Škovierová, Dušan Braný, Erika Halašová, Zuzana Danková

**Affiliations:** 1 Division of Molecular Medicine, Biomedical Center Martin, Jessenius Faculty of Medicine in Martin, Comenius University in Bratislava, 036 01 Martin, Slovakia; 2 Division of Oncology, Biomedical Center Martin, Jessenius Faculty of Medicine in Martin, Comenius University in Bratislava, 036 01 Martin, Slovakia

**Keywords:** liquid biopsy, cell-free nucleic acids, circulating tumor cells, leiomyomas, sarcomas, leiomyosarcomas, exosomes

## Abstract

Utilization of liquid biopsy in the management of cancerous diseases is becoming more attractive. This method can overcome typical limitations of tissue biopsies, especially invasiveness, no repeatability, and the inability to monitor responses to medication during treatment as well as condition during follow-up. Liquid biopsy also provides greater possibility of early prediction of cancer presence. Corpus uteri mesenchymal tumors are comprised of benign variants, which are mostly leiomyomas, but also a heterogenous group of malignant sarcomas. Pre-surgical differentiation between these tumors is very difficult and the final description of tumor characteristics usually requires excision and histological examination. The leiomyomas and malignant leiomyosarcomas are especially difficult to distinguish and can, therefore, be easily misdiagnosed. Because of the very aggressive character of sarcomas, liquid biopsy based on early diagnosis and differentiation of these tumors would be extremely helpful. Moreover, after excision of the tumor, liquid biopsy can contribute to an increased knowledge of sarcoma behavior at the molecular level, especially on the formation of metastases which is still not well understood. In this review, we summarize the most important knowledge of mesenchymal uterine tumors, the possibilities and benefits of liquid biopsy utilization, the types of molecules and cells that can be analyzed with this approach, and the possibility of their isolation and capture. Finally, we review the typical abnormalities of leiomyomas and sarcomas that can be searched and analyzed in liquid biopsy samples with the final aim to pre-surgically differentiate between benign and malignant mesenchymal tumors.

## 1. Introduction

Uterine mesenchymal tumors (UMT) are a very heterogeneous group comprised of benign variants, which are mostly leiomyomas (ULM) and also malignant sarcomas. ULM are very common, affecting over 50% of Caucasian women and up to 80% of African origin women and they are one of the main causes of hysterectomy [1,2]. The incidence may be even higher, particularly in developing countries, because ULM can be asymptomatic in more than 50% of women who are, therefore, unaware of their presence [1,2]. Although they do not primarily endanger patients´ lives, ULM can markedly decrease the quality of life if they become symptomatic [3]. In contrast, sarcomas are very rare, with an estimated incidence of three to seven per 100,000 women [4], comprising only up to 8% of all corpus uteri malignancies [5]. Sarcomas are also very aggressive. They spread rapidly and have a low five-year survival rate and high rates of recurrence with low therapeutic efficacy [5,6,7].

UMT heterogeneity causes complicated classification. The most common form of UMT is “conventional” or “typical” ULM. This covers 90–95% of all lesions and these are considered benign without signs of malignancy [1,3,8]. This group is also heterogeneous, so that “conventional” ULM can differ in the following characteristics: (1) in histological degenerative changes such as myxoid, hyaline, fatty, hemorrhagic, and cystic changes [8]; (2) their localization in the uterus [3]; and (3) the type of genetic driver alternations [9].

In addition to strictly benign ULM forms, the remaining 5–10% can mimic malignancy and, therefore, display some of typical malignant traits such as higher mitotic index, nuclear atypia or the presence of coagulant necrosis [8,10]. There are also smooth muscle proliferations with unusual growth patterns, including benign metastatic ULM or intravenous leiomyomatosis [11]. The most common variants of non-conventional ULM are atypical, bizarre-cellular and mitotically active [8,10]. The most unpredictable, but still considered non-malignant UMT, are the smooth muscle tumors of uncertain potential, abbreviated STUMP [12]. In addition, inflammatory myofibroblastic tumors are quite rare in the uterus, but can closely mimic both benign and malignant variants of UMT [13]. Debate also continues concerning whether uterine leiomyosarcomas (LMS) arise from malignant transformation of ULM [14,15,16], but, herein, we consider ULM and LMS two separate entities, as generally assumed [5].

The malignant sarcoma group is also very heterogeneous; comprising histologically different tumors of various origin. These are divided into nonepithelial and mixed epithelial-nonepithelial categories dependent on the type of cancerous cell and its presumed originating tissue [7]. However, a better explanation of the variability of these tumors is characterized by sarcoma division into the four previously traditional categories: (1) homologous sarcomas composed only of uterine tissue; (2) heterogeneous types with mixed uterine and non-uterine tissues, typically striated muscle, bone, and cartilage; (3) pure types which comprise only mesodermal structures; and (4) mixed types with mesodermal and non-mesodermal structures; mainly epidermal [17]. These categories could have been combined, for example, LMS were categorized as homologous pure tumors. Nonetheless, this traditional classification incorrectly included several mixed mesenchymal tumors such as carcinosarcomas which are currently listed as tumors of endometrial origin [18]. Moreover, the variability of sarcomas means that future reclassification cannot be excluded. According to present-day classification, the most common type of sarcomas are LMS, comprising up to 60%, followed by up to 20% endometrial stromal sarcomas (ESS), up to 10% adenosarcomas, and the remaining 10% comprises other types of sarcomas [19].

UMT heterogeneity makes it very difficult to pre-surgically distinguish particular types without histopathologic confirmation after excision; including diagnosis of mitotic index, coagulant necrosis, and cytological atypia [5,6]. It is still difficult to distinguish mesenchymal lesions, even with the help of standard imaging devices—variable types of ultrasonography, magnetic resonance or computed tomography (1) malignant sarcomas from benign ULM [1,3] and (2) one type of malignant tumor from other types [5], and even when helpful clinical indications, for example, usual sarcomas occurrence and ULM nonoccurrence after menopause are available [20]. Moreover, non-imaging clinical diagnostic approaches also cannot identify UMT type with certainty; these methods are more suited to identifying endometrial lesions [5,21].

Importantly, ULM are especially difficult to differentiate from the LMS [3,5], because imaging and collateral symptoms are very similar; notably those of abnormal uterine bleeding, abdominal and pelvic pain, dysmenorrhea, and urinary problems [1,7]. Furthermore, ULM and LMS have some common features connected with their formation, especially higher ER receptor expression and activity [22], higher incidence in African origin and obese women [2,7], and both tumor types are more frequent in women with diabetes mellitus [23]. The many similarities between ULM and LMS include also their same origin by transformation of the same cells [24]. Therefore, it would be very beneficial to utilize molecular approaches to distinguish between particular UMT types and this could overcome insufficiencies in pre-surgical clinical diagnosis, especially in ULM and LMS [25]. These factors have inspired the main focus of this article on the use of a noninvasive approach to differentiate between these two tumor groups and we also present brief information on other sarcoma types. This mainly includes ESS, which can be differentiated from other UMT types, and, especially, LMS through molecular approaches [26].

## 2. Liquid Biopsy

The presence of tumorous cells released into the bloodstream was first described in the nineteenth century by Thomas Ashworth [27]. This can be considered the discovery of the phenomenon now known as liquid biopsy (LB). In general terms, LB is described as a noninvasive technique which enables analysis of molecular biomarkers sampled from body fluids. While this primarily concerns blood [28], it also includes cerebrospinal fluid, pleural effusion, broncho-alveolar lavage fluid, saliva, urine, and sputum [29]. The main concept of LB is to enable more simple, efficient, faster and less-expensive monitoring of disease status, response to treatment, metastasis progression, and early differentiation of particular tumor types. All this can be achieved by analyzing the body fluid targets which are described in greater detail in the following section. Finally, LB is a revolutionary technique that uses modern and precise medical principles, which can help clinicians in therapeutic decisions and make the diagnostic process less stressful for patients. LB has mainly arisen because of invasive method limitations which include the following: (1) it can have unexpected complications and cannot be performed when clinical conditions have worsened or when a tumor is inaccessible [30], (2) it is often unrepeatable and provides only information on the tumor at one point in time and precludes monitoring responses to medication during both treatment and follow up, (3) it cannot be used for screening or early prediction of cancerous disease, and (4) it is generally performed after incidental early-stage tumor identification or following clinical examination resulting from exacerbated patient symptomatology [28,31]. In addition, many patients fear invasive procedures, prefer noninvasive diagnosis, and medicated non-surgical treatment. Furthermore, over 50% women with UMT want to preserve their uterus and fertility [32].

In contrast, the test results from LB are more suitable for early diagnosis because these are usually available much earlier, they have potential to estimate the risk of metastasis progression and relapse more precisely, and they are usually less expensive [28,31]. Simply taking the blood or other body fluids and analyzing target molecules and cells is, therefore, beneficial in many ways, but despite the above mentioned benefits it is important to realize that LB is often used only for assessment of tumor characteristics, but when the LB diagnoses the tumor as malignant it still requires surgical excision [33,34]. In addition, the detection ability of LB still remains challenging and further studies are required to test its accuracy and ability to correctly identify various tumor types. It also remains uncertain if LB constantly provides a representative sampling of the whole tumor mass or only its parts. However, intratumoral and spatial heterogeneity can also often limit precise interpretation of the molecular profile in tissue biopsies [33]. Finally, there is, in some aspects a lack of established consensus to guide its utilization [33,34] and despite the LB approach being beneficial and becoming more and more popular, in some aspects, confirmation of its precise, advanced, and reliable use in clinical praxis is required.

## 3. Cells and Molecules That Can Be Analyzed from LB Samples

The following cells and molecules can be analyzed using the LB approach: circulating tumor DNA (ctDNA); circulating tumor cells (CTC); exosome vesicles; circulating RNAs, i.e., messenger RNA (mRNA); microRNA (miRNA); long non-coding RNA (lncRNA); and also proteins, peptides, and metabolites [29,31].

### 3.1. ctDNA

The ctDNA is tumor derived and fragmented DNA which should preserve the abnormal molecular pattern present in the primary tumor [35,36,37,38,39]. Nonetheless, some studies also claim that ctDNA’s pattern of abnormalities generally differs from those in primary tumors [40,41,42]. The ctDNA is primarily released from tumors by apoptosis and necrosis (programmed and not programmed cell death), but also by tumor secretion [43]. Various factors affect the total composition of ctDNA circulating in the bloodstream; it is especially dependent on tumor status, burden, and histopathology [44].

### 3.2. CTC

CTC can be valuable disease indicators because they are exfoliated from both primary tumors and metastatic lesions. Their numbers and concentration can be used as a predictive marker, and as with exosomes, they contain variable nucleic acids, proteins, and metabolites which can be utilized in molecular analyses at the mutational, transcriptional, and epigenetical levels [45,46].

### 3.3. Exosomes

Exosomes are membrane-bound phospholipid vesicles, actively secreted by a variety of cells, including cancer cells, and they contain proteins as well as the following nucleic acids: miRNAs, lncRNAs, and mRNAs from tumors. Although the half-life of exosomes in bloodstream is still analyzed and debated, Boukoris et Mathivanan [47] considered exosomes highly stable in stored conditions, and therefore compared to freely circulating nucleic acids (CNA), those molecules encapsulated in exosomes should also be more stable in stored conditions. However, miRNAs, which can also be contained in exosomes, are generally considered very stable and their activity should remain the same, regardless if they are encapsulated in exosomes or incorporated in ribonuclear complexes [48].

### 3.4. mRNAs, miRNAs and lncRNAs

mRNAs, miRNAs and lncRNAs are present in the bloodstream through both active secretion in exosomes and apoptotic/necrotic cell lysis. However, apart from miRNAs, the half-life of other RNAs in the bloodstream is presumed to be very short and their utilization for LB, therefore, requires further analysis [49,50].

All the above elements, however, are very rare. For example, ctDNA comprises only 0.1% to 10% of all circulating free DNA (cfDNA) released into the bloodstream from all human cells. Especially leucocytes [51] and cfDNA concentration is itself quite low at 10 to 100 ng/mL [52]. The ctDNA concentration, therefore, ranges from 0.01 ng/mL to 10 ng/mL and while the half-life of unencapsulated particles ranges usually from 15 minutes to 2.5 h [31,53], ctDNA half-time is considered relatively long, i.e., at least two hours [51].

In addition, CTC have a frequency as low as 1 CTC per 10^6^–10^7^ leucocytes which is usually less than 1 CTC per milliliter of blood [46]. Therefore, it is necessary to sample higher blood volumes, usually ranging from 5 to 9 mL to detect at least one CTC. Their occurrence also depends on the tumor stage, where lower numbers are more common in early-stage disease and this increases only slightly in patients with advanced metastases [54,55].

Rajagopal et al. [56] considered that increased exosome number is a potent biomarker for abnormal physiological states and that their total numbers increase in cancer patients as compared to healthy subjects. However, precise exosome quantification is difficult to determine because, in addition to total number, the variations in size, and protein and nucleic acid content, per vesicle can vary and “quantification” of these latter variations should be considered in further molecular analysis [57].

## 4. Capture, Enrichment, and Isolation of Freely Circulating Vesicles, Molecules and Cells 

Traditional exosome capture and enrichment includes ultrafiltration and size exclusion chromatography, precipitation with polymers, and immunoaffinity purification by magnetic beads [58]. These methods can be utilized because exosomes have specific biological patterns, and their cellular origin provides typical surface markers. These include the CD9, CD63, and CD81 members of the tetraspanin family, heat-shock proteins such as HSP70, and the Rab protein family [59]. However, the exosome surface protein profile can vary with both the character and origin of the cell that released them, and therefore establishing more specific membrane biomarkers and the genetic profile of exosomes would be beneficial for using exosomes as agents in novel targeted therapies [60].

Microfluid-based systems are now becoming more popular than traditional exosome capturing systems, because these can directly analyze blood samples and are, therefore, more practical for clinical praxis [45]. However, regardless of the capture technique used, it is very important to the utilization of exosomes in clinical diagnostics, and potentially also in tumor differentiation, that the nucleic acids and metabolites encapsulated in these vesicles can be analyzed following the electron microscope validation and isolation [60,61]. While most current analyzes have targeted the expression of RNA transcripts, which are abundantly present in these exosomes [61,62], there is scant knowledge of the genomic and epigenetic profile of DNAs encapsulated in these vesicles.

ctDNA isolation is relatively easier than capturing exosomes. The main ctDNA isolation limiting factor is its separation from other cfDNA molecules, however, both of the following targeted and untargeted approaches are now available for this separation [36].

(1) Targeted approaches are based on identifying the following mutations/abnormalities in the ctDNA elements: (a) previously accepted abnormal patterns in ctDNAs (e.g., in different cancer type or based on *in silico* prediction) and (b) the same pattern in the primary tumor and the released ctDNA. Here, the “standard” methods of molecular biology such as variable PCR modifications and pyrosequencing can be used [63,64,65,66], but precise and more sensitive methods including BEAMing, droplet digital PCR, and next generation sequencing (NGS) are more suitable because of the very low ctDNA levels as compared to cfDNAs [67,68,69].

(2) Nontargeted approaches require no prior knowledge of molecular alternatives and while these are generally based on NGS techniques, the digital karyotyping and PARE methods can also be utilized [36]. Whereas, both targeted and nontargeted approaches are used mainly for detecting point mutations, insertions and deletions, amplifications, translocations, and copy number alterations [36,67]; identifying abnormal epigenetic patterns and especially changes in promoter region methylation levels can be a suitable alternative to genomic alterations. It is supposed that ctDNA epigenetic patterns should also usually remain the same as in the tumors from which they were released and also be specific for both cancer type and progression [35,67,70,71,72,73,74,75].

The capture of CTC remains extremely challenging because of its rarity and short half-life. There are still affinity-based methods used which take advantage of antigens that are differentially expressed by CTC [46]. These include: (1) epCAM which is normally present on the surface of cells released from epithelial carcinomas [45,46]; (2) the MUC1 present in breast and ovary carcinomas [76,77]; and (3) the EphB4 which is typically over-expressed in advanced head and neck cancers [46]. However, this approach remains specific for limited cancer types because these markers are suitable for positive selection, and therefore knowledge of further CTC surface antigens typical for different cancer types is essential. A further limitation is imposed by epithelial cells which are undergoing epithelial-mesenchymal transition (EMT) and cannot be captured by positive selection [45,46]. Negative selection is used to avoid selection bias and is based on capturing and subsequent removal of non-CTC cells in the blood, most importantly, leucocytes. These are commonly depleted through immunomagnetic targeting and removal against CD45 and other leucocyte antigens [45,78]. To the best of our knowledge, no studies have focused on identifying CTC released from any type of uterine sarcoma or ULM. However, there are some options for the identification of CTC from different non-uterine sarcoma types, and this provides opportunities to determine CTC from uterine sarcomas. For example, Benini et al. [79] were able to identify CTC released from Ewing sarcomas by immunoseparation with CD99 antibody and magnetic microbeads. The origin of the cells was verified by the presence of fusions typical for this tumor. The expression of cell-surface vimentin provided an even better chance to identify CTC of various sarcomatous origins [80]. Most recently, Hayashi et al. [81] recorded CTC identification from various sarcoma types based on positive vimentin staining, negative CD45 staining, and nuclear morphology distinct from normal white blood cells.

The standard approaches for identifying CTC include using magnetic beads armed with antibodies for positive or negative separation and employing columns or cartridges or microchips coated with antibodies [82]. However, the CTC physical properties, such as electric charges, can also be used to enrich and isolate these cells with more practical microfluid cell sorters [83,84] or Microhall platforms [85,86]. Finally, microfiltration systems, such as CellSieve^TM^, also provide great possibilities for the identification of variable CTC with only minimal cell processing [81]. Interestingly, CTC were previously thought to be released only from metastases, but it is now accepted that two CTC “types” exist. This is contrary to previous claims, because the first type acts as a metastatic factor and the other is only passively released into the bloodstream, and although it should not promote metastases formation, its role in tumor spread remains undetermined [87].

## 5. Known Abnormalities in UMT

LB methodology enables analysis of various molecular targets over longer periods and this makes it possible to monitor disease progression and response to treatment. Furthermore, it also provides the possibility of differentiating particular tumor types. This can be very beneficial for early diagnosis of cancer. For differentiation of UMT with the LB approach, there are two options: (1) assessment of the mutational/transcriptional/epigenetic pattern in solid tumors and subsequent identification of the very same abnormalities in LB samples and (2) determination of specific patterns of change in the candidate markers directly in the LB samples and their connection with particular cancer diseases.

Unfortunately, specific LB biomarkers with the potential to non-invasively distinguish particular UMT types have not been assessed. Further analysis is, therefore, required to find a successful marker, but this is quite promising because the presence of ctDNA released from ULM and malignant sarcomas has been noted in non-invasive prenatal testing (NIPT) [88,89,90]. Furthermore, Hemming et al. [91,92] described the presence of ctDNA from LMS in LB samples and these correlated with tumor progression. Moreover, many of the biomarkers of solid UMT described below have previously been reported in LB from patients with different cancer diseases and these provide the possibility of early diagnosis, prognosis, and therapeutic outcome. These include the identical microRNAs [93,94,95,96,97], methylation changes [95,98,99], mutational changes [100,101,102], and even similar chromosomal rearrangements [103,104,105,106]. We, therefore, suggest a way to identify LB biomarkers suitable for UMT differentiation by searching for abnormal patterns already established in solid UMT in LB samples. This possibility is very promising also because reports identify that solid tumor abnormalities should remain unchanged in CNA [35,36,37,38,39,67,70,71,72,73,74,75] and eventually also in vesicles and CTC. Therefore, all abnormal patterns listed below are noted in solid tumors and their summary can form the basis for designing LB-based analysis in patients with UMT.

## 6. miRNAs

The miRNAs are short non-coding RNAs which can bind to target mRNAs and induce gene silencing and repression of transcription [107]. The 2000 miRNA genes already identified in the human genome is expected to increase in number and their abnormal activity has been connected with the development of various cancerous diseases because they regulate the important cellular functions of development, differentiation, growth and metabolism [107]. Analysis of miRNA abnormal activity is currently attractive, however, in ULM and LMS tissues, it is still not sufficiently known. The most notable abnormally expressed microRNAs in ULM and LMS tissues are listed in Table 1. There are some interesting characteristics of miRNAs in these tumors including that treatment with 17β-oestradiol and medroxyprogesterone acetate should result in the regulation of variable miRNAs’ activity, at least in the ULM and LMS cell lines [108]. Liu et al. [109] noted that various miRNAs are differentially active in African and Caucasian women. Wang et al. [110] considered that the abnormalities in microRNAs activity should be associated with tumor size as well as ethnicity. Moreover, ULM typically have an abnormal extracellular matrix, and miR-15b is more active in ULM tissue samples than in healthy myometrium (MM), and also more active in ULM cell lines than in MM cell lines [111]. This miRNA is supposed to regulate RECK protein expression in normal physiological status to ensure negative regulation of matrix metalloproteinases [112,113]. In addition, the let-7 family miRNA family has the *HMGA2* gene as a target and *HMGA2* abnormal expression, especially from translocations, is considered a typical ULM mark [114].

The activity of most analyzed miRNAs has been assessed in ULM. MicroRNA activity is less understood in LMS and studies that could compare the activity of identical microRNAs in both tumor types are widely lacking. Exceptions, however, include studies by de Almeida et al. [115] and Schiavon et al. [116], which focused on assessing miRNA activity in both tumor types. In the former study, the authors identified 24 oncomirs with de-regulated expression profile in both ULM and LMS. The latter work recorded similar results in establishing widely different ULM and LMS expression patterns. Danielsson et al. [117] also suggested that different miRNA activity should contribute to separate ULM and LMS development, and they created a clustering system differentiating ULM and LMS based on miRNA activity. Unfortunately, however, they accomplished this only in cell lines. The most notably different ULM and LMS miRNAs recorded in these studies are listed in Table 1, under the “observed in LMS/ULM tissue” section, and these can be considered the best candidates for distinguishing between ULM and LMS on miRNA activity using LB. However, many of the analyzed ULM miRNAs were not analyzed in malignant LMS, even in the two separate studies. Therefore, the miRNAs summarized in Table 1 and reported as abnormal in ULM (listed under the “observed in ULM/MM tissues”), could prove beneficial if analyzed in LMS. There are also studies which assessed different miRNAs expression profiles in ESS. For example, Kowalewska et al. [118] reported four miRNAs which were differentially active in ESS and the control group, but it is quite interesting that they noted no significant differences in miRNA expression in LMS and control healthy tissues. The additional study by dos Anjos et al. [119] compared LMS, carcinosarcoma, and ESS miRNA profiles and identified both variable miRNA activity in these three tumor types and a variety of miRNAs connected with lower cancer-specific survival rates. This may, however, be due to tumor differences, whereas LMS are primarily smooth-muscle tumors. ESS, even of mesenchymal origin, are mixed tumors with a large proportion of endometrial elements and carcinosarcomas are classified as being of endometrial origin [18].

## 7. DNA Mutations and Chromosomal Aberrations in ULM and Sarcomas

ULM often result from gene mutations, but this occurs only on a small scale. The most mutated is the *MED12* gene observed in almost 70% of ULM, and the second most frequent is *FH* gene mutations which comprise only approximately 1% [129,130]. In addition, non-sporadic germline mutations of this gene occurred more often in women with both ULM and LMS [131,132]. Interestingly, malignant uterine LMS also have *MED12* gene mutation in almost 70% of tumors [133], and this can be, therefore, considered a typical mark for these tumors. While this common mutation potentially distinguishes the two tumor groups from healthy tissue, unfortunately it cannot be used as a distinguishing factor of these tumors and is, therefore, unsuitable for LB diagnostics. In contrast to ULM, LMS have quite often mutated *TP53* gene [6,134]. Although mutations in this gene are typical for many cancerous diseases, LB determination of *TP53* mutation in women with previous image-diagnosed UMT should always provoke rapid tissue biopsy, subsequent histological examination, and close observation of the patient. Moreover, this gene should also be considered a potential candidate for gene therapy in women with LMS [135].

The *ATRX* gene is also relatively often mutated in LMS tissues [134], and also in STUMP [136]. Furthermore, the *RB1* gene is typically mutated in LMS [26] and in ULM with bizarre nuclei [137]. However, the percentage of mutations of both genes in LMS is less than 50% and the extent of *ATRX* mutation in leiomyomatous tissues remains undetermined. In addition, ULM with bizarre nuclei are quite rare and the *RB1* gene is often mutated in a wide range of cancers [138]. In summary, the mutation profiles of ULM and sarcomas are not well understood, and currently demonstrated abnormalities provide only limited possibilities for LB differentiation of these tumors. However, mutation changes in the *TP53*, *ATRX,* and *RB1* genes have been detected and analyzed in LB samples in patients with different cancer diseases [100,101,102]. In contrast to mutational changes, the ULM and LMS chromosomal aberrations are quite different and better understood, so current knowledge suggests that LB differentiation of these tumors based on chromosomal aberrations is more suitable than just relying on mutational changes. Moreover, this identification from LB samples is feasible, for example, with ultra-low passage whole genome sequencing [91,139,140]. The most important recognized chromosomal aberrations in ULM and LMS are listed in Table 2.

## 8. Methylation Changes in ULM and LMS

The methylation profiles of ULM and sarcoma promoter regions provide further possibilities for tumor differentiation based on LB, because changes in DNA methylation patterns can be identified in ctDNA and have been proposed as potential biomarkers for tumor staging, prognosis, and monitoring of the treatment response [31,98,162]. It has also been suggested that abnormal methylation patterns should remain identical in both the primary tumors and the ctDNAs released by the tumor [35,67,70,71,72,73,74,75]. Maekawa et al. [163] reported great variability in ULM methylation patterns. These authors reported 120 genes with different methylation pattern in ULM and MM, and 22 of these were estrogen receptor alpha (ERα) target genes, thus, indicating that their abnormal methylation could contribute to abnormal estrogen response. One of these genes is *DAPK1* which is often abnormally methylated in various cancer types [164] and importantly, it has also been reported abnormally methylated in the serum of women with ULM [165].

Following bisulfide-sequencing of the ERα receptor gene promoter region, Asada et al. [166] reported that seven CpGs in distal sites are often variably methylated in healthy and tumorous tissues. It is quite likely that methylation variations in distal ERα regions are dependent on menstrual cycle phases [167], and since this has already been assessed in solid tissues, it would be very interesting and beneficial to analyze the status in LB samples. ULM also have differentially methylated sex chromosomes [168] where the most notably hypomethylated was the *TSPYL2* gene. In addition, women of African origin have hypermethylated *DLEC1*, *KRT1,9* and *KLF11* genes in ULM as compared to MM [169].

Importantly, methylation markers have already been used by Sato et al. [170] to distinguish between ULM and LMS, especially by establishing a hierarchical clustering system able to discriminate between these tumors with 70% accuracy. The system is based on different methylation levels in ten genes. This study is also unique because it assesses the methylation status in both ULM and LMS tumor types. In contrast, the remaining methylation markers listed in Table 3 were assessed either for benign or malignant lesions, but not for both, and further analyses comparing the status of these genes in both tumor types would be beneficial to determine how well they coud distinguish between these tumor types. For LMS are typical aberrations altering the cell cycle [6], whereby promoter region hypermethylation is supposed to induces loss of function of the important cell cycle regulator *CDKN2* [171] and hypermethylation also most likely causes the *BRCA1* gene´s protein reduction in almost 30% of uterine LMS [172].

Malignant LMS have abnormal estrogen receptor activity and sensitivity, but these are lower than in benign ULM [22]. The LMS also have often hypomethylated *ESR1* target genes [26] and the polycomb group target genes [173]. Importantly, authors Miyata et al. [173] claimed that ULM and LMS can be distinguished by their global DNA methylation levels, and also presumed that the methylation pattern is different on a genome-wide level in ULM subtypes with different genetic driver aberration, i.e., *MED12* mutation, *HMGA2* activation, and *FH* mutation [9,174]. These results make tumor differentiation based on their ctDNA methylation levels very interesting, however, the bloodstream ctDNAs are very fragmented and poorly represented, and therefore ctDNA methylation analyses on either a genome-wide scale or a locus-specific level should preferably use very sensitive novel sequencing approaches [67,98]. The hyper- and hypomethylated genes in ULM and LMS tissues are listed in Table 3.

## 9. lncRNAs and mRNAs

ULM expression profiles are relatively well assessed in studies analyzing gene activity on the mRNA levels in solid tumors [25,178,179,180]. The LMS mRNA expression profile is also relatively sufficiently known [6,25,181,182] and variably expressed genes are recognized also in primary and metastatic LMS [183] and in LMS as comparing to ESS [184]. Although mRNA molecules directly released into the bloodstream after apoptosis or necrosis can technically reflect intracellular processes in tumor cells and their levels can have some predictive value of tumor character, their very short half-life, instability, low abundance, and regular contamination with other intracellular mRNAs severely limits them as useful biomarkers [185]. In contrast, assessment of mRNA expression in CTC and exosomes appears more suitable because of the newly developed approaches [186,187,188,189].

Genome-wide analyses of ULM lncRNA expression have demonstrated that hundreds of these molecules are aberrantly active in ULM as compared to MM [190]. The number of abnormally active lncRNAs correlates with tumor size, and lncRNA H19 was considered as the most important abnormally active lncRNA in ULM [190]. lncRNA H19 contributes to *MED12* and *HMGA2* gene regulation. It is assumed that lncRNA H19 should also affects the activity of various extracellular matrix remodeling genes [191]. Therefore, further study of this lncRNA should shed more light on the regulation of important ULM marker expression and this lncRNA could potentially be used as a reliable distinguishing marker of these tumors. Finally, further research of lncRNAs’ abnormal activity in rare malignant sarcomas should also prove advantageous, but this is still currently lacking.

## 10. ULM and Sarcoma Metabolites

ULM are also the subject of metabolic studies because of their high occurrence, the search for efficacious medication, and their great current cost. The very interesting study performed by Heinonen et al. [192] assessed abnormal metabolomes and metabolic pathways in ULM, and they associated these with particular molecular subtypes, which harbor different driver genetic abnormalities. These were divided into the following four groups: those with the mutated *MED12* gene, those with *HMGA2* gene translocations, the group with *FH* gene biallelic inactivation, and *COL4A5*-*COL4A6* deletions [9]. In summary, Heinonen et al. [192] recorded 70 dysregulated metabolites in all ULM types, and the characteristic dysregulations in particular ULM subtypes included: (1) the FH subtype which has characteristic metabolic alterations in the tricarboxylic acid cycle and pentose phosphate pathways and increased levels of multiple lipids and amino acids and (2) the MED12 subtype which has markedly reduced levels of vitamin A and dysregulation of ascorbate metabolism. Interestingly, the retinoid acid receptor pathways have previously been observed to be very dysregulated in ULM, and this caused lower levels of biologically active retinoid acid and increased levels of its abnormal metabolites [9,193,194]. In addition, it has been suggested that premenopausal parous women with ULM share a wide range of metabolic syndrome features such as higher serum triglycerides levels (TG ≥ 150 mg/dL), low serum-high density lipoproteins (<50 mg/dL), and hyperglycemia (FPG ≥ 100 mg/dL) [195]. The obesity and high blood pressure observed in this syndrome have also been previously and repetitively associated with ULM occurrence [1,2,3]. However, while it is generally accepted [3] that vitamin D deficiency is strongly associated with ULM occurrence, its abnormal metabolites were not confirmed in these tumors [192].

The uterine sarcoma metabolite profile is poorly understood due to its rare occurrence. Paradoxically, some authors regard metabolomic markers more reliable than molecular markers in sarcoma diagnostics, especially in the high-grade type which are more difficult to distinguish histologically. Moreover, individual tumors often contain areas of different histologic grade, necrotic regions, and variable inflammatory cell infiltrate, and this heterogeneity hinders the search for molecular markers [196,197]. However, the preference for metabolomic markers over the molecular remains disputed and subjective.

Lastly, the same pattern of precursor ion differences has been found in LMS, myxofibrosarcomas, and undifferentiated pleiomorphic sarcomas. The two metabolite signals correlate with overall survival, *m/z* 180.9436 and *m/z* 241.0118; and one with metastasis-free survival, *m/z* 160.8417. In addition, FTICR-MSI identified *m*/*z* 241.0118 as inositol cyclic phosphate and *m*/*z* 160.8417 as carnitine [197].

## 11. Discussion and Conclusions

In this review, we based the differentiation of UMT on the LB approach because of the diverse action of non-coding RNAs, and mutational and methylation changes and differences in metabolic activity in ULM, other UMT, and MM. We also suggested searching for these previously known solid tumor abnormalities in LB samples. However, the differentiation of two or more tumor types by LB necessitates, in the final step, assessment of their altered patterns in the body fluid samples. For example, hypermethylation or mutational changes can be considered proof of ULM rather than LMS when a LB sample is abnormally hypermethylated or has particular mutations, and this abnormal pattern was previously observed with high predictive value in ULM but not in LMS. This explanation, however, is simplistic and follows only basic principles because the entire procedure for establishing a particular marker to its utilization in clinical testing is a long and multistep process. While it is debatable if there is sufficient knowledge of the differences in ULM, it is certainly lacking in sarcomas. Therefore, to enable LB to determine tumor character and to monitor the response to treatment and tumor progression in malignant variants, it is essential to increase current knowledge of changes in ULM and also to perform these analyses in uterine sarcomas, especially LMS.

Moreover, we based our suggestions on the assumption that changes in CNA should remain the same as they are in the primary tumors which release them [35,36,37,38,39,67,70,71,72,73,74,75]. However, it should be mentioned, some studies suggest that the changes can vary [40,41,42]. In addition, it is necessary to realize that metastatic lesions can differ from primary tumors in their molecular patterns [183]. It would, therefore, be beneficial to perform more comparative analyses of primary and metastatic sarcomas to understand the changes in their progression. In addition, it would also be helpful to use more gene assays with high predictive value in LB screening, instead of sole ones. Finally, there is still no successful tool for differentiating UMT, so even achieving lower predictive value would be advantageous. An example here is that assays correctly assessing tumor type in eight out of 10 women would be better than none.

It is quite intriguing that there is no interest in developing techniques that could differentiate such frequent tumors as ULM and such aggressive tumors as sarcomas. The main reason appears to be the very low incidence of sarcomas and most UMT lesions are ULM. These latter are not life threatening and often asymptomatic. In addition, the statistical threat to life caused by uterine sarcomas is low, and almost negligible in comparison to the “deadliest” cancer types such as breast, lung and colon cancer. However, if sarcomatous lesions are primarily misdiagnosed as ULM, this can have fatal consequences, especially when women refuse surgical excision and prefer medicated treatment. Moreover, although uterine sarcomas are rare, these lesions still affect thousands of women globally and can be easily misdiagnosed. Therefore, early noninvasive diagnosis could provide certainty that lesions are really benign; and it could significantly prolong full-quality life even when the lesion is malign. Moreover, appropriate LB approaches can monitor both the metastatic progression of malignant lesions and response to treatment, and thus contribute to their better understanding.

Unfortunately, minimal analyses have focused directly on capturing and identifying circulating elements from UMT and most findings have been incidental and collateral. It is known that NIPT results can be affected by the presence of tumorous circulating elements [88]. The first study of these discrepancies we are aware of was conducted by Osoborne et al. [198]. Woman with metastatic neuroendocrine carcinoma gave birth to a healthy infant, and although both invasive testing during pregnancy and postnatal placental histology revealed no abnormalities, NIPT testing indicated trisomy in chromosomes 13 and 18. These trisomes, therefore, arose from release of tumor DNA into the bloodstream and not due to infant chromosomal aberrations. A summary of a three-year period of NIPT testing enabled identification of 55 samples with altered genomic profiles and thus not reportable NIPT results [89]. All these discrepancies are presumed to arise due to the presence of various neoplasm types, but importantly almost half of the identified discrepancies arose as a result of the presence of benign ULM and only one because of uterine sarcomas presence.

One study focused directly on identifying circulating ctDNAs from uterine LMS and confirmed ctDNA presence in five of 10 cases [91]. This number is considered quite low, but sarcomatous ctDNA was identified in five of six patients with progressive disease. Therefore, ctDNA identification correlated with tumor burden, and it was present in patients with both primary and metastatic lesions but not identified in disease-free or stable subjects. This was a pilot study, so repetition and finding new approaches and targets should increase the numbers of positively identified LMS ctDNAs.

In addition, Eastley et al. [199] confirmed the possibility of detecting and subsequently analyzing ctDNAs released from soft tissue tumors, including two leiomyosarcoma samples. The authors found *TP53* and *HRAS* mutations in both primary tumors and plasma samples. However, it is necessary to remember that the percentage of these mutations differs in tissue and plasma samples and the authors do not mention if these leiomyosarcomas arise from uterine tissues or not.

The understanding of CTC and exosome release from ULM and sarcomas is still poor, and to the best of our knowledge no miRNA profiles have been recovered from these tumors in LB samples despite their abundance in primary tumors. More studies should, therefore, focus on assessing non-coding RNAs, especially miRNAs in LB samples and on the character of CTC and exosomes released from UMT. From a positive perspective, however, combined approaches have already been developed for the purposes of LB-based tumor diagnosis and prognosis, and these detect desired ctDNA changes in mesenchymal tumors, including uterine LMS [200].

In conclusion, there are no currently known LB biomarkers that can be used for early and noninvasive distinction between particular UMT types. However, (1) there are recorded nucleic acid molecules released from both benign and malignant variants into the bloodstream [89,90,91,92]; (2) many molecular biomarkers reported in solid UMT have been noted in LB samples in different cancer types and these can be used for early diagnosis, prognosis, and therapeutic outcomes [93,94,95,96,97,98,99,100,101,102]; and (3) it is presumed that abnormal patterns in solid tumors should remain the same in CNA [35,36,37,38,39,67,73,74,75]. On the basis of these facts, we can state that it is theoretically possible, and highly likely in practice, to distinguish clinically between UMT types using the LB approach when appropriate molecular markers are employed. Our review should, therefore, inspire further research and analysis of known abnormalities in solid tissues in LB samples. The implication of the markers in these processes could also help monitoring of both the progression of malignant variants and the response to treatment. It is, therefore, essential in the future to establish as many molecular markers as possible for each type of UMT and to evaluate them using LB approaches. This will be most beneficial when sufficiently large patient cohort screening can be performed with a variety of advanced molecular methods. Performing NGS-targeted approaches for identification of the markers highlighted in this review is suggested as a most suitable option. Moreover, genome-wide studies would also be welcome to identify useful markers, including epigenetic markers. Finally, any possible improvements in LB approaches for women with UMT will benefit both patients and clinicians, because clinicians would then be able to more precisely measure disease burden and better coordinate clinical treatment, providing greater certainty to affected women that their lesion remains benign. 

## Figures and Tables

**Table 1:** Abnormally active microRNAs in ULM and LMS.

ULM			LMS		
miRNA	Observed in	Expression in Tumorous Tissue	miRNA	Observed in	Expression in Tumorous Tissue
Let-7 family * [110]	ULM/MM tissue	Up	miR-15a * [120]	Primary/metastatic LMS tissue	Up in metastases
miR-27a * [110]	ULM/MM tissue	Up	miR-92a * [120]	Primary/metastatic LMS tissue	Up in metastases
miR-21 * [110]	ULM/MM tissue	Up	miR-31 * [120]	Primary/metastatic LMS tissue	Up in primary
miR-23b * [110]	ULM/MM tissue	Up	miR-122-5p [116]	LMS/ULM tissue	Up
miR-200a * [121]	UtLM-hTert	Up	miR-206 * [116]	LMS/ULM tissue	Up
miR-542-3b [122]	ULM/MM tissue	Up	miR-373-3p * [116]	LMS/ULM tissue	Up
miR-377 [122]	ULM/MM tissue	Up	miR-144-3p [116]	LMS/ULM tissue	Up
miR-363 [123]	ULM/MM tissue	Up	miR-372-3p * [116]	LMS/ULM tissue	Up
miR-490 * [123]	ULM/MM tissue	Up	miR-34a-5p [116]	LMS/ULM tissue	Down
miR-137 [123]	ULM/MM tissue	Up	miR-27b-3p [116]	LMS/ULM tissue	Down
miR-15b * [111]	ULM/MM tissue; ULM/MM cell lines	Up	miR-135b-5p [116]	LMS/ULM tissue	Down
miR-30a [121]	ULM/MM tissue; UtlM-hTERT cell lines	Up	miR-9-5p [116]	LMS/ULM tissue	Down
miR-32 [110]	ULM/MM tissue	Down	miR-10b-5p * [115]	LMS/ULM tissue	Up
miR-29b * [110,124]	ULM/MM tissue	Down	miR-125b-1-3p [115]	LMS/ULM tissue	Up
miR-542-5p [122]	ULM/MM tissue	Down	miR-140-5p * [115]	LMS/ULM tissue	Up
miR-642 [122]	ULM/MM tissue	Down	miR-145-5p [115]	LMS/ULM tissue	Up
miR-93/106 [125]	ULM/MM tissue	Down	miR-130b-3p * [92]	LMS/ULM tissue	Down
miR-486-5p * [123]	ULM/MM tissue	Down	miR-148-3p [115]	LMS/ULM tissue	Down
miR-217 [123]	ULM/MM tissue	Down	miR-204-5p [92]	LMS/ULM tissue	Down
miR-4792 [123]	ULM/MM tissue	Down	miR-203a-3p [92]	LMS/ULM tissue	Down
miR-200a * [109]	ULM/MM tissue; UtlM-hTERT cell lines	Down	miR-152 * [126]	LMS/ULM tissue /SKLMS1cell lines	Down
mir-143 * [108]	ULM/MM tissue	Down			
miR-200c * [127]	ULM/MM tissue	Down			
miR-197 * [128]	ULM/MM tissue	Down			
miR-212 [121]	ULM/MM tissue; UtlM-hTERT cell lines	Down			

* These were reported and analysed in LB samples in patients with different cancer diseases [93,94,95,96,97]; ULM—uterine leiomyomas, MM—healthy myometrium, LMS—uterine leiomyosarcomas.

**Table 2:** Most typical chromosomal rearrangements in ULM, LMS, and ESS.

Chromosomal Aberrations in ULM			Chromosomal Aberrations in LMS			Chromosomal Aberrations in ESS		
Chromosome/Locus	Type	Affected Genes	Chromosome/Locus	Type	Affected Genes	Chromosome/Locus	Type	Affected Genes
12q15 [141]	Translocation	*HMGA2*	12q13-15 [142]	Amplification	*RB1*	(7;17)(p15;q21) [143]	Translocation	*JAZF1,SUZ12*
14q24 [144]	Translocation	*RAD51B*	10q21.3 * [145]	Loss	*PTEN*	(6;7)(p21;p15) [146]	Translocation	*JAZF1-PHF1*
7(q22q32) [147]	Deletion	*CUX1*	13q14.2-q14.3 * [145]	Loss	*LEU*	(6;10)(p21;p11) [146]	Translocation	*EPC1-PHF1*
6p21 [148]	Translocation	*HMGA1*	7q36.3 [142]	Gain	*PTPRN2*	(1;6)(p34;p21) [149]	Translocation	*MEAF6-PHF1*
10q22 [150]	Translocation	*KAT6B*	7q33-q35 [142]	Gain	*HAVCR1*	(X;17)(p11;q21) [151]	Translocation	*CXorf67-MBTD1*
1(q31q43) [130]	Deletion	*FH*	1p21.1 * [142]	Loss	*AMY2A*	(X;22)(p11;q13) [152]	Translocation	*ZC3H7B-BCOR*
Xq22 [153]	Deletion	*COL4A5*/*COl4A6*	9p.21	Gain	*CDKN2*	(10;17)(q22;p13) [154]	Translocation	*YWHAE-NUTM*
1p36 [155]	Translocation/deletion	*AJAPI, NPHP*	12q.15 [156]	Gain	*MDM2*	der(22)t(X;22)(p11;q13) [152]	Deletion	/
8q12 [157]	Insertion/translocation	*PLAG1*	1q21 * [158]	Amplification	*FLF, PRUNE*	del(16)(q22) [149]	Deletion	/
19q * [159]	Deletion	/	5p14-pter [158,160]	Amplification	/			
12 [161]	Trisomy	/	13q31 [158,160]	Amplification	/			
10 [147]	Monosomy	/	19p13 [158,160]	Amplification	/			
22q * [159]	Deletion/monosomy	/	20q13 [158,160]	Amplification	/			

* Similar chromosomal rearrangements were reported in LB samples from patients with different cancer diseases [103,104,105,106]; ULM—uterine leiomyomas, LMS—uterine leiomyosarcomas, ESS—endometrial stromal sarcomas.

**Table 3:** Abnormally methylated genes in ULM and LMS.

ULM		LMS	
Gene	Methylation Change	Gene	Methylation Change
*IRS1* [163]	Hypermethylation	*MGMT* * [175]	Hypermethylation
*COL4A1* [163]	Hypermethylation	*BRCA1* * [172]	Hypermethylation
*GSTM5* [163]	Hypermethylation	*CDKN2* * [137]	Hypermethylation
*DAPK1* * [165]	Hypermethylation	*PTEN* [6]	Hypermethylation
*KLF11* [169]	Hypermethylation	*RASSF1A* *^a^ [176]	Hypermethylation
*DLEC1* * [169]	Hypermethylation	*DAPK1* *^a^ [177]	Hypermethylation
*KRT19* [169]	Hypermethylation		
*ALX1* ‡ [170]	Hypermethylation		
*CBLN1* ‡ [170]	Hypermethylation		
*CORIN* ‡ [170]	Hypermethylation		
*DUSP6* [170]	Hypermethylation		
*FOXP1* ‡ [170]	Hypermethylation		
*GATA2* ‡* [170]	Hypermethylation		
*IGLON5* ‡ [170]	Hypermethylation		
*NPTX2* ‡* [170]	Hypermethylation		
*NTRK2* ‡ [170]	Hypermethylation		
*STEAP4* ‡* [170]	Hypermethylation		
*PRL* ‡ [170]	Hypomethylation		
*PART1* [170]	Hypomethylation		
*TSPYL2* [168]	Hypomethylation		
*OCRL* [168]	Hypomethylation		

* These were reported and analyzed in LB samples in patients with different cancer diseases [95,98,99]; ‡ Methylation status of these genes was compared between ULM and LMS in a study by Sato et al. [170]; ^a^ Methylation changes were noted in variable types of leiomyosarcomas, including non-uterine. *DAPK1* gene was reported as hypermethylated in ULM and sarcomas as compared with healthy tissues, but it was not compared between ULM and LMS; ULM—uterine leiomyomas, LMS—uterine leiomyosarcomas.

## Abbreviations

cfDNA	circulating free DNA
CNA	freely circulating nucleic acids
CTC	circulating tumor cells
ctDNA	circulating tumor DNA
EMT	epithelial-mesenchymal transition
ERα	estrogen receptor alpha
ESS	endometrial stromal sarcomas
LB	liquid biopsy
LMS	uterine leiomyosarcomas
lncRNA	long non-coding RNA
mRNA	messenger RNA
miRNA	microRNA
MM	healthy myometrium
NGS	next generation sequencing
NIPT	non-invasive prenatal testing
STUMP	smooth muscle tumors of uncertain malignant potential
ULM	uterine leiomyomas
UMT	uterine mesenchymal tumors
